# Clinical Efficacy and Safety of Misoprostol During Abdominal Myomectomy: An Updated Systematic Review and Meta-Analysis of 16 Randomized Controlled Trials

**DOI:** 10.3390/jcm13216356

**Published:** 2024-10-24

**Authors:** Ahmed Abu-Zaid, Maha Al Baalharith, Mohannad Alsabban, Osama Alomar, Mohammed Abuzaid, Saud Owaimer Alsehaimi, Hedaya Albelwi, Saad M. S. Alqarni, Manal Ali Alqahtani, Mohammed Ziad Jamjoom, Saeed Baradwan, Hussein Sabban, Samah Himayda, Bayan Albouq, Ehab Badghish, Afnan Baradwan, Raghad Sindi, Ismail A. Al-Badawi

**Affiliations:** 1 College of Medicine, Alfaisal University, Riyadh 11533, Saudi Arabia; 2 Department of Obstetrics and Gynecology, King Abdulaziz Medical City, Riyadh 11426, Saudi Arabia; 3 Department of Obstetrics and Gynecology, King Faisal Specialist Hospital and Research Center, Riyadh 11211, Saudi Arabia; 4 Department of Obstetrics and Gynecology, Al Birk General Hospital, Al Birk 63525, Saudi Arabia; 5 Department of Obstetrics and Gynecology, National Guard Hospital, Riyadh 14611, Saudi Arabia; 6 Department of Obstetrics and Gynecology, King Faisal Armed Forces Hospital, Khamis Mushait 62413, Saudi Arabia; 7 Department of Obstetrics and Gynecology, King Faisal Medical City for Southern Regions, Abha 62527, Saudi Arabia; 8 Department of Obstetrics and Gynecology, King Fahad Armed Forces Hospital, Jeddah 23311, Saudi Arabia; 9 Department of Obstetrics and Gynecology, King Faisal Specialist Hospital and Research Center, Jeddah 23433, Saudi Arabia; 10 Department of Obstetrics and Gynecology, Faculty of Medicine at Rabigh, King Abdulaziz University, Jeddah 21589, Saudi Arabia; 11 Department of Obstetrics and Gynecology, Faculty of Medicine, Umm Al-Qura University, Makkah 21955, Saudi Arabia; 12 Department of Obstetrics and Gynecology, Prince Mohammed Bin Abdulaziz National Guard Hospital, Madinah 42324, Saudi Arabia; 13 Department of Obstetrics and Gynecology, Maternity and Children Hospital, Makkah 24246, Saudi Arabia; 14 Department of Obstetrics and Gynecology, Al Salama Hospital, Jeddah 22233, Saudi Arabia; 15 Faculty of Pharmacy, Umm Al-Qura University, Makkah 24381, Saudi Arabia

**Keywords:** fibroids, misoprostol, myomectomy, bleeding, meta-analysis

## Abstract

**Objective**: This study offered an updated meta-analysis of randomized controlled trials (RCTs) that assessed preoperative misoprostol compared to control (matched placebos or no treatment) during abdominal myomectomies. **Methods**: Six databases underwent screening until 7 April 2024. The risk of bias was assessed using the Cochrane Collaboration tool. The results were presented as mean differences (MDs) or risk ratios (RRs) along with 95% confidence intervals (CIs) using the random-effects model. **Results**: Sixteen RCTs were analyzed, involving 975 women. The overall quality of the studies was rated as “low” or had “some concerns” of bias in seven and eight RCTs, respectively; one RCT had an overall “high” risk of bias. For primary endpoints, the misoprostol intervention had significantly lower mean intraoperative blood loss (n = 15, MD = −180.2 mL, 95% CI [−224.04, −136.35], *p* < 0.001), mean hemoglobin drop (n = 13, MD = −0.58 g/dl, 95% CI [−0.82, −0.35], *p* < 0.001), and rate of perioperative blood transfusion (n = 13, RR = 0.43, 95% CI [0.29, 0.63], *p* < 0.001) compared to the control intervention. For secondary endpoints, the misoprostol intervention had significantly lower mean hematocrit drop (MD = 2.15, 95% CI −3.34, −0.96], *p* < 0.001), mean operative time (MD = −12.95 min, 95% CI [−19.89, −6.01], *p* < 0.001), and mean hospital stay (MD = −0.14 days, 95% CI [−0.25, −0.02], *p* = 0.02) compared to the control intervention. Nonetheless, no significant change was indicated between both interventions regarding the rate of postoperative fever. **Conclusions**: During abdominal myomectomy, the administration of preoperative misoprostol was generally safe and yielded statistically significant reductions in intraoperative blood loss, hemoglobin drop, and perioperative blood transfusion.

## 1. Introduction

Uterine fibroids stand as the commonest noncancerous growths in the female reproductive system globally. Although they frequently develop without noticeable symptoms, around one-third of individuals encounter symptomatic occurrences, leading to a variety of notable challenges. These can entail pelvic discomfort, increased menstrual flow, anemia due to iron deficiency, difficulties with fertility, and complications in pregnancy [1,2].

Myomectomy is the foremost option to consider when conservative treatment fails to effectively alleviate symptoms. Additionally, for patients aiming to retain their uterus for fertility and future childbearing, myomectomy emerges as the preferred surgical option [1,2].

Uterine fibroids, known for their rich vascularity, carry a significant risk of intraoperative bleeding during myomectomy [3]. Furthermore, myomectomy inherently involves risks, with perioperative blood loss being a frequently reported complication [4,5]. The intraoperative blood loss can be substantial enough to lead to life-threatening consequences, including hemodynamic instability due to anemia, the necessity for blood transfusions, and potentially fatal outcomes if not appropriately managed [4]. Hence, it is crucial to implement strategies aimed at reducing bleeding and its related aftermaths during myomectomy to decrease both the disease burden and death risks.

A growing pool of research has examined the clinical utility of different perioperative pharmacological intermediations designed to lessen intraoperative bleeding and its morbidities during myomectomy. In this context, misoprostol, a man-made prostaglandin E1 analogue known for its uterotonic and vasoconstrictive properties [6,7], has gained recognition for its hemostatic effects in postpartum hemorrhage [8] and hysterectomy [9]. From a mechanistic standpoint, misoprostol can manifest antihemorrhagic effects during myomectomy through two primary processes. The first process is ascribed to its uterotonic effects, which enhance myometrial contractions, leading to the contraction of vascular structures and subsequently reducing blood flow [6,7,10]. The second process is credited to its direct vasoconstrictive effects on uterine vasculature, culminating in diminished blood flow [10,11].

In 2021, Wali and colleagues conducted an analysis of eight randomized controlled trials (RCTs) involving 385 patients to assess the safety and clinical utility of misoprostol versus placebo in open myomectomies [10]. The researchers concluded that misoprostol reduced both intraoperative bleeding and the requirement for blood transfusion, without an increase in postoperative fever [12]. Since then, eight further RCTs have been published with conflicting findings and constrained by small sample sizes [13,14,15,16,17,18,19,20]. This underscores the necessity to thoroughly synthesize all available evidence (totaling 16 RCTs) and offer comprehensive, robust, up-to-date recommendations regarding the clinical utility and toxicity of preoperative misoprostol in abdominal myomectomies.

Therefore, this research was geared to upgrade the work performed by Wali et al. in 2021 [12] and carried out an updated meta-analysis of 16 RCTs that assessed preoperative misoprostol compared to control (matched placebos or no treatment) during abdominal myomectomies.

## 2. Methods

### 2.1. Study Protocol

We completed this unregistered investigation following the steps outlined in the Preferred Reporting Items for Systematic Reviews and Meta-Analyses (PRISMA) statement [21]. Furthermore, ethical approval was unnecessary as our study exclusively relied on published literature.

### 2.2. Eligibility Criteria

We analyzed studies meeting the following conditions: (i) women undergoing abdominal myomectomy, (ii) experimental intervention involving preoperative misoprostol, (iii) control intervention comprising matched placebo or nothing given, (iv) studies reporting at least one of the pre-determined primary endpoints of the investigation, including intraoperative blood loss, hemoglobin drop, or rate of perioperative blood transfusion, and (v) study design as RCTs. We excluded investigations that did not meet these criteria, including non-randomized studies, studies involving non-abdominal approach of myomectomy (e.g., laparoscopy), studies using combinational drugs with misoprostol (e.g., vasopressin), and studies comparing misoprostol with an active pharmacologic treatment (e.g., oxytocin) or intervention (e.g., uterine artery tourniquet).

### 2.3. Literature Search

We searched for literature across six electronic databases, comprising Cochrane Central Register of Controlled Trials, Google Scholar, PubMed, Web of Science, Scopus, Embase, and PubMed. Database screening included records from inception until 7 April 2024. The precise search method is delineated in Supplementary Table S1 and comprised the following query: (PGE1 OR “prostaglandin E1” OR misotac OR cytotec OR misoprostol) AND (myomectomy) AND (open OR abdominal OR laparotomy). No filters, including language, publication date, or geography, were applied during literature search. Two investigators independently accomplished the database search and resolved any discrepancies through agreement.

### 2.4. Study Selection

After eliminating duplicates, we evaluated titles along with abstracts to exclude irrelevant citations. Then, a thorough examination of the full texts determined the final selection of relevant studies. To ensure no applicable studies were overlooked, we inspected the reference lists of included studies and recent review articles. Two investigators independently selected the studies and resolved any discrepancies through agreement.

### 2.5. Data Gathering and Study Endpoints

We gathered baseline characteristics of the analyzed studies and their subjects, which encompassed author names, publication dates, countries of publication, study arms, participant sample sizes, participant ages, participant body mass indices, number of myomas, size of the largest myomas, types of uterine fibroids, and details of misoprostol and control interventions. The primary endpoints comprised mean intraoperative blood loss (mL), mean hemoglobin drop (g/dL), and rate of perioperative blood transfusion. The secondary endpoints comprised mean hematocrit drop (%), mean operation time (min), mean hospital stay (day), and rate of postoperative fever. Three teams, each containing two investigators, independently extracted the data, rectifying any inconsistencies through agreement within each team.

### 2.6. Evaluation of Study Quality

We evaluated the quality of the analyzed RCTs using the related updated (version 2) Cochrane risk of bias assessment tool [22]. Two investigators independently conducted the evaluation of study quality, rectifying any inconsistencies through mutual agreement.

### 2.7. Evaluation of Certainty of Evidence

We evaluated the certainty of evidence according to the GRADE (Grading of Recommendations, Assessment, Development, and Evaluations) approach. Two investigators independently conducted the evaluation of certainty of evidence, rectifying any inconsistencies through mutual agreement.

### 2.8. Data Synthesis

STATA (StataCorp LLC, College Station, TX, USA), version 18.0 for Windows, was used for all statistical analyses. Whenever applicable, we imputed standard deviations for changes from baseline using the steps emphasized in the Cochrane Handbook for Systematic Reviews of Interventions [23]. After applying forest plots, we reported the continuous data as mean differences (MDs) and dichotomous data as risk ratios (RRs), accompanied by 95% confidence intervals (CIs), in a random-effects model by DerSimonian and Laird [24]. Assessment of between-study heterogeneity was conducted based on Higgin’s I^2^ statistic (>50%) and the chi-square test (*p* < 0.1) [25]. Sensitivity analyses (leave-one-out) were conducted by sequentially excluding one RCT at a time to assess the robustness of the summary results. Publication bias was assessed using funnel plots and Eggers’ regression test for outcomes with at least 10 RCTs [26]. Subgroup meta-analysis was performed according to the route of misoprostol administration for the heterogenous outcomes. Statistical significance for primary and secondary endpoints was defined as a two-tailed *p*-value < 0.05.

## 3. Results

### 3.1. Summary of Study Selection and Baseline Characteristics

**Figure 1:**
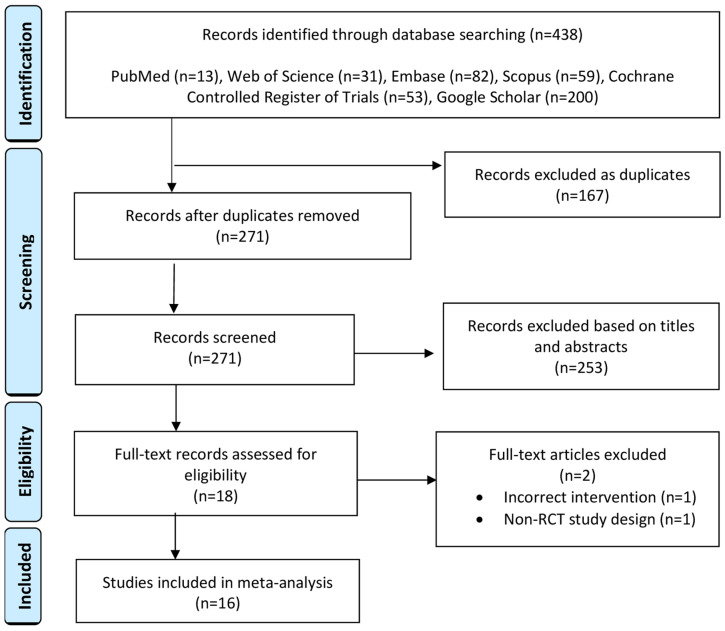
Summary of the PRISMA flow diagram for database screening and study selection.

Figure 1 presents a summary of the PRISMA flow diagram for database screening and study selection. At the full-text stage, 18 citations were screened. One citation was excluded because it incorrectly reported the intervention; the study included patients who underwent both laparoscopic and abdominal myomectomy without providing separate data for each [27]. The second citation, sourced from Google Scholar, appeared to be a retrospective study, lacked abstract and complete bibliographical information (i.e., journal title, volume, issue, and page numbers), and was not accessible in full text. Collectively, 16 RCTs were analyzed, consisting of 975 patients: 487 in the misoprostol group and 488 in the control group [13,14,15,16,17,18,19,20,28,29,30,31,32,33,34,35]. Table 1 presents a summary of the baseline characteristics of the analyzed studies and their subjects. These RCTs, conducted between 2005 and 2023, took place in various countries, including Egypt (n = 7), Iran (n = 3), Pakistan (n = 2), Uganda (n = 1), Turkey (n = 1), Nigeria (n = 1), and Thailand (n = 1). The sample sizes ranged from 13 to 63 patients in the misoprostol group and from 12 to 63 patients in the control group. The route of misoprostol administration varied, including rectal (n = 8 RCTs), vaginal (n = 6 RCTs), and sublingual (n = 2 RCTs). These misoprostol doses were administered 0.5 to 3 h preoperatively. The control treatments included a matched placebo in 11 RCTs, no treatment in four RCTs, and intravenous saline in one RCT. No significant variations were noted between the groups regarding age, body mass index, number of myomas, and the size of the largest myoma. The site of the myoma was not reported in eight RCTs.

### 3.2. Summary of Study Quality

**Figure 2:**
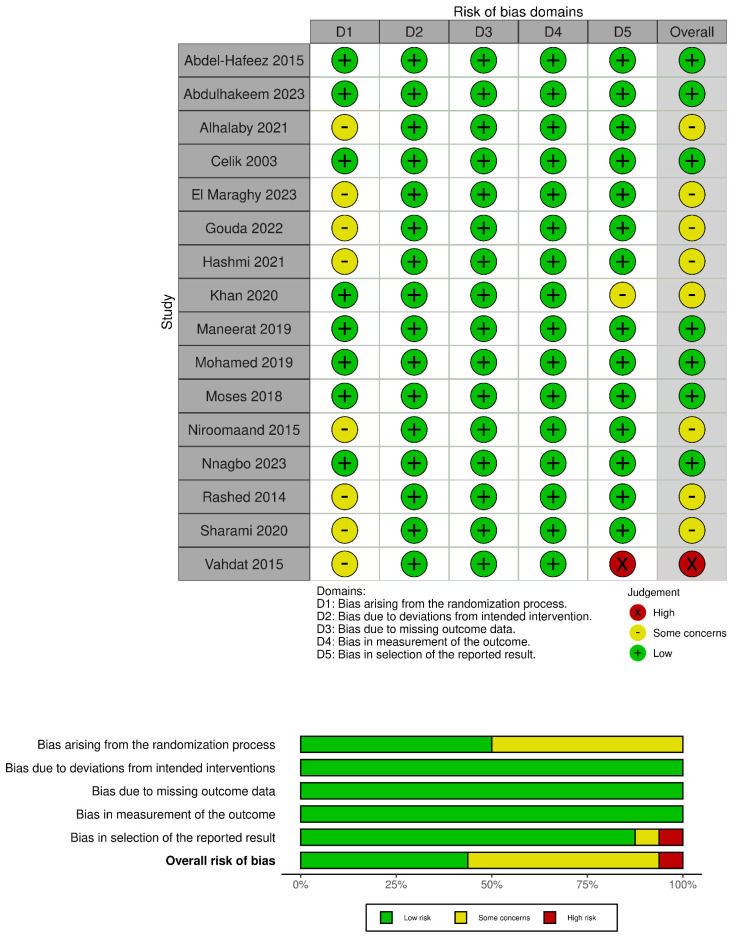
Summary of the quality of the included studies.

Figure 2 presents a summary of the quality of the included studies. The overall quality of the RCTs varied: seven had a low risk of bias, eight had some concerns regarding bias, and one had a high risk of bias. Several studies (n = 8) did not offer ample information about allocation concealment [13,16,17,18,19,29,33], leading to concerns about bias in the randomization process. In one study, the primary outcome of intraoperative blood loss was not reported, resulting in a high risk of bias for the selection of the reported result [15]. In another study [34], the reported outcomes were missing standard deviations, leading to concerns about bias in the selection of the reported result.

### 3.3. Summary of Meta-Analysis of the Primary Endpoints

**Figure 3:**
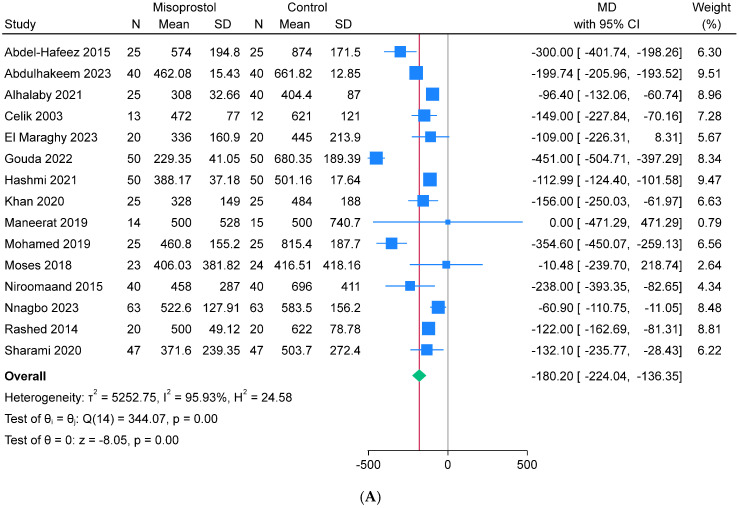

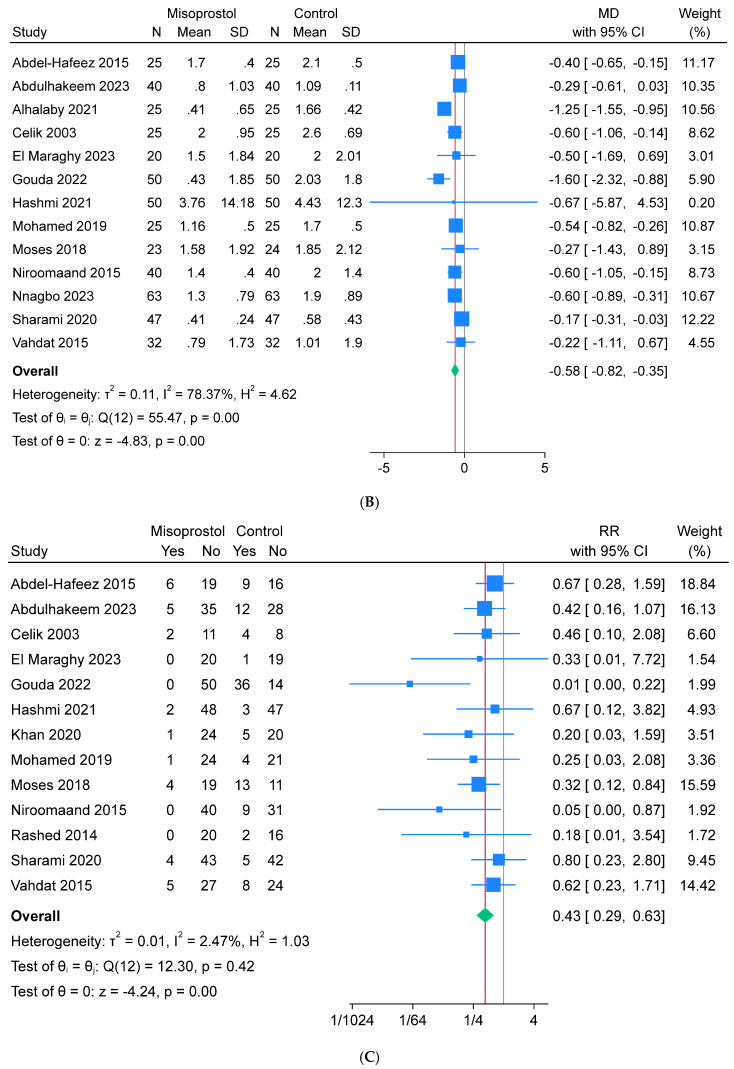
Meta-analysis of the primary endpoints: (**A**) intraoperative blood loss (mL), (**B**) mean hemoglobin drop (g/dL), and (**C**) perioperative blood transfusion (%).

The misoprostol intervention had significantly lower mean intraoperative blood loss (n = 15, MD = −180.2 mL, 95% CI [−224.04, −136.35], *p* < 0.001), mean hemoglobin drop (n = 13, MD = −0.58 g/dL, 95% CI [−0.82, −0.35], *p* < 0.001), and rate of perioperative blood transfusion (n = 13, RR = 0.43, 95% CI [0.29, 0.63], *p* < 0.001) compared to the control intervention. The analyses depicted significant heterogeneity for the intraoperative blood loss (I^2^ = 95.93%) and hemoglobin drop (I^2^ = 78.37%), but homogeneity for the perioperative blood transfusion (I^2^ = 2.47%) (Figure 3A–C).

### 3.4. Summary of Meta-Analysis of the Secondary Endpoints

**Figure 4:**
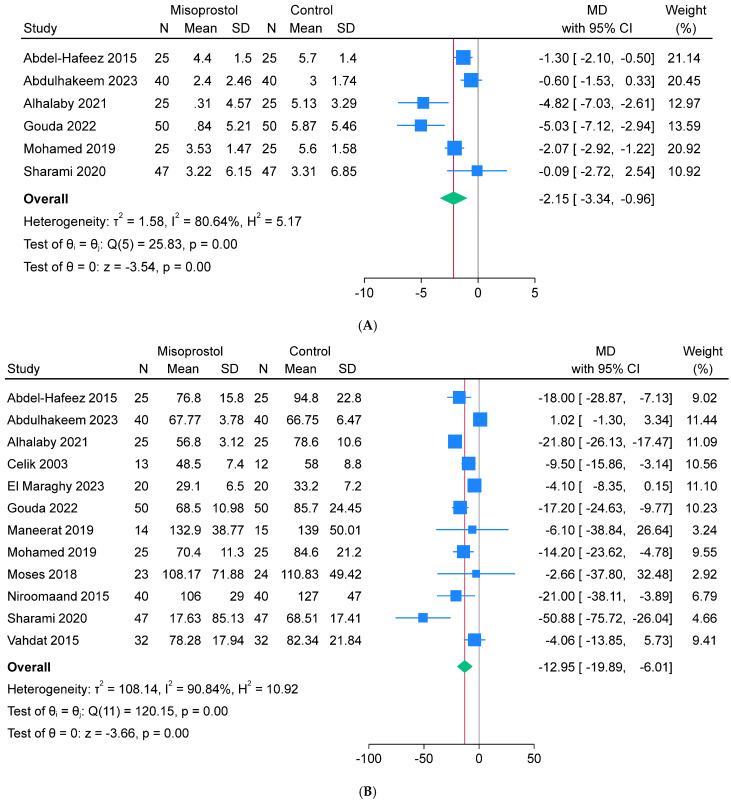

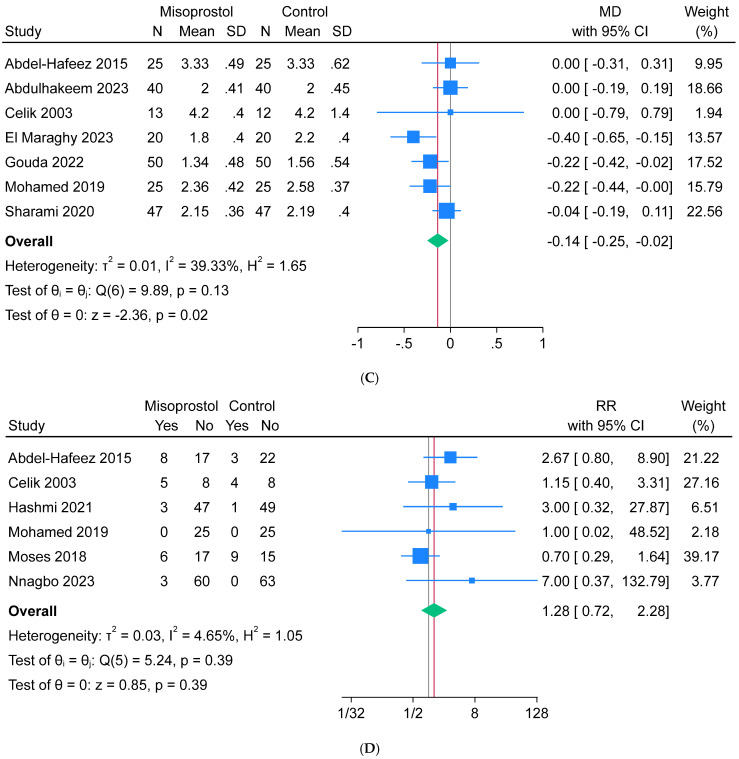
Meta-analysis of the secondary endpoints: (**A**) hematocrit drop (%), (**B**) operative time (min), (**C**) hospital stay (days), and (**D**) postoperative fever (%).

The misoprostol intervention had significantly lower mean hematocrit drop (n = 6, MD = 2.15%, 95% CI −3.34, −0.96], *p* < 0.001), mean operative time (n = 12, MD = −12.95 min, 95% CI [−19.89, −6.01], *p* < 0.001), and mean hospital stay (n = 7, MD = −0.14 days, 95% CI [−0.25, −0.02], *p* = 0.02) compared to the control intervention. The analyses exhibited significant heterogeneity for the hematocrit drop (I^2^ = 80.64%) and operative time (I^2^ = 90.84%), but homogeneity for the hospital stay (I^2^ = 39.33%) (Figure 4A–C). On the other hand, no significant change was indicated between both interventions regarding the rate of postoperative fever (n = 6, RR = 1.28, 95% CI [0.72, 2.28], *p* = 0.39), and the analysis depicted homogeneity (I^2^ = 4.65%) (Figure 4D).

### 3.5. Summary of Subgroup Meta-Analysis, Sensitivity Analysis, Publication Bias, and Certainty of Evidence

Supplementary Figure S1 presents a subgroup meta-analysis based on the route of misoprostol administration for the heterogeneous outcomes, but it did not resolve the heterogeneity completely. All outcomes demonstrated robustness during the leave-one-out sensitivity analysis, except for the mean hospital stay, where omitting some studies altered the summary effect sizes (Supplementary Figures S2 and S3). No evidence of publication bias was noted for all primary endpoints and the operative time endpoint, as indicated by the lack of funnel plot asymmetry (Supplementary Figure S4) and non-significant Egger’s regression test results (all *p* > 0.1, Supplementary Table S2). The certainty of evidence was “moderate” for most endpoints (Supplementary Table S3).

## 4. Discussion

This meta-analysis sought to update the evidence on the role of preoperative misoprostol during abdominal myomectomy. Sixteen (n = 16) RCTs were analyzed, involving 975 women (487 receiving misoprostol and 488 in the control group). The overall quality of the studies was rated as “low” or had “some concerns” of bias in seven and eight RCTs, respectively; one RCT had an overall “high risk” of bias. Compared to the control intervention, the findings revealed that misoprostol administration led to statistically significant reductions in intraoperative blood loss, hemoglobin drop, and perioperative blood transfusion. Additionally, misoprostol significantly reduced hematocrit drop and operative time. The rate of postoperative fever did not significantly differ between the two interventions. Most endpoints exhibited significant heterogeneity. Data robustness was confirmed for all endpoints during sensitivity analysis, except for hospital stay. No evidence of publication bias was detected for the evaluated outcomes. The certainty of evidence was “moderate” for most outcomes and ranged from “low” to “high”.

Uterine fibroids have been identified as hypoxic tumors [36], harboring a microenvironment that fosters increased angiogenesis [3]. As a result, the increased vascularity of uterine fibroids may drastically boost the risk of intraoperative bleeding during myomectomy. Myomectomy can be accomplished through traditional abdominal surgery or less invasive techniques, such as laparoscopy, hysteroscopy, or robot [37]. In low-resource settings, abdominal myomectomy is the most prevalent approach, yet it also carries the highest bleeding-related morbidity rate [38]. Managing blood loss effectively during this procedure is essential, given that substantial intraoperative hemorrhage can lead to several untoward aftermaths. These comprise reduced visibility within the operative field and increased vulnerability to surgeon-induced injuries. Additionally, there is a higher probability of experiencing significant complications such as hemodynamic instability, requiring blood transfusions, and infections that may impede wound healing [39]. Thus, patients undergoing abdominal myomectomy may benefit from preoperative administration of hemostatic drugs such as misoprostol, which can help mitigate associated risks. Our results demonstrate the efficacy and safety of misoprostol in this context. Additionally, similar findings have been observed in patients undergoing minimally invasive myomectomy procedures [27,40,41,42].

In surgical procedures like abdominal myomectomy, it is justified to employ drugs to improve hemostasis, whether administered systemically or topically [43,44]. Instances of such drugs encompass tranexamic acid (an antifibrinolytic agent) [45], vasopressin (a vasoconstrictor agent) [46], and oxytocin (a uterotonic agent) [47]. In addition to these agents, misoprostol has become increasingly popular as an antihemorrhagic medication in women-related procedures [8,9]. Given the potential adverse effects associated with systemic agents like misoprostol, oxytocin, or vasopressin, the use of locally active agents, such as topical hemostats like gelatin-thrombin matrix, should be considered [48]. Watrowski et al. investigated the effectiveness of a gelatin-thrombin matrix in 83 patients compared to 132 control patients undergoing major gynecological and gynecologic-oncological procedures [48]. The authors found that the use of the gelatin-thrombin matrix led to significant reductions in intraoperative bleeding, operative time, and postoperative hemoglobin drop. The study concluded that the gelatin-thrombin matrix was associated with markedly improved short-term perioperative outcomes. Due to its local action, this hemostatic agent appears especially beneficial for patients who cannot pause anticoagulant medication.

Misoprostol can be administered through assorted routes, such as orally, sublingually, buccally, rectally, and vaginally. Eight out of the 16 RCTs utilized the rectal route (50%). The vaginal and rectal routes show comparable onset of action, typically within 20 min, with peak concentrations achieved within 1 h. The drug-related effects of both routes can last for up to 4–6 h [6]. However, the vaginal route has demonstrated higher bioavailability compared to the rectal one [6,49]. It is important to acknowledge that absorption through the vaginal route can vary, as it may be influenced by the quantity and pH (acidity/basicity) level of vaginal discharge among patients [6]. Furthermore, the vaginal route may be more practically and socially suitable to patients compared with the rectal one. When misoprostol is administered orally or sublingually, it reaches its peak concentration earlier (about 30 min) and then rapidly declines to very low levels after a short duration (about 2 h) [49,50,51].

Overall, misoprostol is widely recommended by the World Health Organization for numerous obstetric and gynecologic uses due to its notable efficacy, safety, affordability, stability at room temperature, and widespread availability in many countries [52]. These attributes are particularly advantageous and warranted in low-resource settings. In our limited analysis of safety for postoperative fever, misoprostol exhibited a safe profile compared to the control intervention, which further reinforces the clinical value of misoprostol use for hemostasis during abdominal myomectomy.

Due to its aforementioned encouraging pharmacokinetic [6,49,50,51] and pharmacodynamic facets [6,7,10,11], misoprostol can be beneficial in mitigating excessive bleeding. High-quality data from meta-analyses articulated the safety and clinical efficacy of misoprostol-related hemostatic effects in postpartum hemorrhage [8] and hysterectomy [9]. Our present meta-analysis extends the clinical applicability of preoperative misoprostol during abdominal myomectomy. Our findings suggest that misoprostol is largely safe and notably diminishes intraoperative blood loss, decreases postoperative hemoglobin and hematocrit drops, and shortens operation duration. Furthermore, misoprostol effectively reduces the requirement for blood transfusions. These results are particularly important in low-resource settings lacking readily accessible blood bank facilities. Moreover, considering the scarcity of blood products and the associated risks of transfusions [53], the utilization of misoprostol becomes even more invaluable.

In 2021, Wali and colleagues conducted a related analysis of eight RCTs involving 385 patients to evaluate the safety and utility of misoprostol versus placebo in open myomectomies [10]. The researchers concluded that misoprostol decreased intraoperative bleeding and the requirement for blood transfusion, without an increase in postoperative fever (n = 4 RCTs) [12]. Our current investigation incorporated an additional eight RCTs, resulting in a larger number of analyzed studies (n = 16) and patients (n = 1000), thereby enhancing the statistical power of the pooled conclusions. Furthermore, we conducted a comprehensive analysis encompassing a broader range of efficacy and safety outcomes. Additionally, we incorporated supplementary analyses to assess the stability of conclusions, publication bias, and certainty of evidence of each outcome (i.e., GRADE approach) [54]; all of which were not conducted previously.

Our study has several shortcomings that need acknowledgment. Certain outcomes demonstrated heterogeneity, potentially stemming from variations in perioperative factors, like patient characteristics, clinical features of uterine fibroids, and routes/doses of drug administration. These influences could indirectly impact the conclusions drawn from the pooled outcomes. Furthermore, we did not investigate publication bias for outcomes with less than 10 RCTs [26]. Many RCTs took place in Egypt, limiting the generalizability and broader perspective of our findings. Additionally, some eligible RCTs raised concerns regarding allocation concealment, potentially undermining the credibility of the conclusions.

Prospective research could explore differential efficacy of preoperative misoprostol based on different surgical techniques employed during myomectomy (e.g., abdominal vs. laparoscopy vs. hysteroscopy) and routes of administration. Additionally, dose-response studies of misoprostol could be conducted to determine the dose associated with optimal efficacy and safety. Head-to-head efficacy/safety and cost-effectiveness analyses could also be performed to compare misoprostol against other commonly active pharmacologic agents (e.g., tranexamic acid) or mechanical methods (e.g., uterine artery tourniquet). Moreover, forthcoming studies could explore the potential synergistic effectiveness of combining misoprostol with other active pharmacologic agents to further mitigate intraoperative bleeding and associated complications during myomectomy. Finally, an intriguing area of research would involve identifying which women undergoing myomectomy are most likely to derive the greatest benefit from the use of misoprostol, based on clinical and fibroid-related features (for example, number and size of myomas).

## 5. Conclusions

During abdominal myomectomy, the administration of preoperative misoprostol was generally safe and appeared to yield significant reductions in intraoperative blood loss, hemoglobin drop, and perioperative blood transfusion. Additionally, misoprostol significantly reduced hematocrit drop and operative time. The rate of postoperative fever and length of hospital stay did not significantly differ between the two interventions. However, the data should be interpreted with caution considering the limitations of the study, namely outcome heterogeneity from perioperative variations, lack of publication bias assessment for outcomes with fewer than 10 RCTs, limited generalizability due to a high number of Egyptian studies, concerns about allocation concealment, and the absence of PROSPERO registration. Additional high-quality investigations are warranted to corroborate this evidence.

## Figures and Tables

**Table 1:** Summary of the baseline characteristics of the analyzed studies and their subjects.

Study Identifier	Country	Study Arms	n	Intervention	Age (Year)	Body Mass Index (kg/m^2^)	Number of Myomas	Size of Largest Myoma (cm)	Site of Myoma
Abdel-Hafeez 2015 [30]	Egypt	Misoprostol	25	Rectally 400 µg, 1 h preop	40.7 ± 5.1	28.9 ± 4.40	2 to 5	14.9 ± 3.95	Intramural, subserous
		Control	25	Matched placebo	40.7 ± 5.5	27.2 ± 5.0	2 to 5	15.9 ± 3.41	
Abdulhakeem 2023 [20]	Egypt	Misoprostol	40	Vaginally 400 µg, 2 h preop	36.53 ± 4.12	22.96 ± 2.36	1 to 3	5 ± 1.48	Not reported
		Control	40	No treatment given	34.62 ± 4.65	23 ± 2.68	1 to 3	4.87 ± 2.12	
Alhalaby 2021 [19]	Egypt	Misoprostol	25	Vaginally 400 µg, 1 h preop	32.88 ± 3.73	25.60 ± 4.11	NR	6.72 ± 1.06	Not repored
		Control	25	No treatment given	34.64 ± 4.10	27.60 ± 4.30	NR	6.64 ± 1.41	
Celik 2003 [28]	Turkey	Misoprostol	13	Vaginally 400 µg, 1 h preop	31.7 ± 4.4	28.3 ± 1.3	5.5 ± 1	15.07 ± 2.86	Intramural, subserous
		Control	12	Matched placebo	32.2 ± 2.9	28.5 ± 1	5.3 ± 0.9	15.42 ± 2.47	
El Maraghy 2023 [18]	Egypt	Misoprostol	20	Sublingually 400 µg, 1 h preop	33.9 ± 5.2	23.09 ± 2.6	Single (n = 16)	3.38 ± 1.52	Submucous
		Control	20	Matched placebo	30.6 ± 5.5	23.8 ± 3.7	Single (n = 16)	2.4 ± 1.36	
Gouda 2022 [17]	Egypt	Misoprostol	25	Rectally 400 µg, 1 h preop	31.72 ± 4.66	NR	Maximum 5	NR	Not reported
		Control	25	Intravenous normal saline	33.82 ± 3.76	NR	Maximum 5	NR	
Hashmi 2021 [16]	Pakistan	Misoprostol	50	Rectally 400 µg, 0.5 h preop	32.16 ± 9.44	26.07 ± 10.44	NR	NR	Not reported
		Control	50	Matched placebo	32.48 ± 7.44	26.55 ± 9.44	NR	NR	
Khan 2020 [15]	Pakistan	Misoprostol	25	Rectally 800 µg, 1 h preop	32.4	NR	Multiple (n = 25)	6.91	Intramural, subserous, submucous
		Control	25	Matched placebo	32.8	NR	Multiple (n = 25)	5.47	
Maneerat 2019 [31]	Thailand	Misoprostol	14	Rectally 400 µg, 0.5 h preop	35.9 ± 5.30	22.3 ± 3.89	NR	NR	Intramural, subserous
		Control	15	Matched placebo	36.3 ± 5.18	22.9 ± 2.95	NR	NR	
Mohamed 2019 [32]	Egypt	Misoprostol	25	Rectally 400 µg, 1 h preop	36.64 ± 3.7	25.7 ± 1.8	1 to 5	11 ± 3.9	Intramural, subserous
		Control	25	Matched placebo	36.7 ± 2.8	25.6 ± 1.8	1 to 5	11.25 ± 3.63	
Moses 2018 [35]	Uganda	Misoprostol	23	Vaginally 400 µg, 1 h preop	35.3 ± 5.36	NR	6.43 ± 4.98	NR	Not reported
		Control	24	No treatment given	34.2 ± 6.19	NR	5.62 ± 4.16	NR	
Niroomandd 2015 [29]	Iran	Misoprostol	40	Vaginally 200 µg, 3 h preop	35.3 ± 6.1	NR	NR	8.7 ± 4.6	Intramural, subserous
		Control	40	Matched placebo	33 ± 5.1	NR	NR	8 ± 2.8	
Nnagbo 2023 [14]	Nigeria	Misoprostol	63	Vaginally 400 µg, 1 h preop	34.1 ± 4.30	27.32 ± 4.27	9.1 ± 3.79	NR	Not reported
		Control	63	No treatment given	33.4 ± 4.44	26.40 ± 3.79	9.41 ± 4.15	NR	
Rashed 2014 [33]	Egypt	Misoprostol	20	Rectally 400 µg, 1 h preop	30.60 ± 2.72	28.10 ± 0.83	NR	NR	Not reported
		Control	20	Matched placebo	31.75 ± 2.45	28.31 ± 0.78	NR	NR	
Sharami 2022 [13]	Iran	Misoprostol	47	Rectally 400 µg, 1 h preop	39.34 ± 6.38	38 ± 4.89	1 to 5	4.6 ± 1.89	Intramural
		Control	47	Matched placebo	25.82 ± 2.89	25.99 ± 3.26	1 to 5	5.31 ± 1.67	
Vahdat 2015 [34]	Iran	Misoprostol	32	Sublingually 200 µg, 0.5 h preop	35.75 ± 5.45	28.37 ± 2.99	2.75 ± 0.71	7.76 ± 1.84	Not reported
		Control	32	Matched placebo	35.69 ± 6.75	28.64 ± 3.14	2.59 ± 0.71	7.25 ± 1.92	

Age and body mass index were reported as mean ± standard deviation (minimum–maximum) or median (minimum–maximum).

## Data Availability

The original contributions presented in the study are included in the article, further inquiries can be directed to the corresponding authors.

## Supplementary Materials

The following supporting information can be downloaded at: https://www.mdpi.com/article/10.3390/jcm13216356/s1.
