# Supplementary material for: Clinical Efficacy and Safety of Misoprostol During Abdominal Myomectomy: An Updated Systematic Review and Meta-Analysis of 16 Randomized Controlled Trials
Source: J Clin Med. 2024 Oct 24;13(21):6356. doi: 10.3390/jcm13216356 (PMC11546417; doi:10.3390/jcm13216356)

Supplementary Figure S1. Subgroup meta-analysis according to the route of misoprostol administration: **[A]** intraoperative blood loss (ml), **[B]** mean hemoglobin drop (g/dl), **[C]** perioperative blood transfusion (%), **[D]** mean hematocrit drop (%), and **[E]** operative time (min).

[A]

Study	Misoprostol N	Misoprostol Mean	Misoprostol SD	Control N	Control Mean	Control SD	MD with 95% CI	Weight (%)
**Rectal**								
Abdel-Hafeez 2015	25	574	194.8	25	874	171.5	-300.00 [-401.74, -198.26]	6.30
Gouda 2022	50	229.35	41.05	50	680.35	189.39	-451.00 [-504.71, -397.29]	8.34
Hashmi 2021	50	388.17	37.18	50	501.16	17.64	-112.99 [-124.40, -101.58]	9.47
Khan 2020	25	328	149	25	484	188	-156.00 [-250.03, -61.97]	6.63
Maneerat 2019	14	500	528	15	500	740.7	0.00 [-471.29, 471.29]	0.79
Mohamed 2019	25	460.8	155.2	25	815.4	187.7	-354.60 [-450.07, -259.13]	6.56
Rashed 2014	20	500	49.12	20	622	78.78	-122.00 [-162.69, -81.31]	8.81
Sharami 2020	47	371.6	239.35	47	503.7	272.4	-132.10 [-235.77, -28.43]	6.22
Heterogeneity: τ^2 = 18051.02, I^2 = 96.07%, H^2 = 25.47							-223.39 [-325.27, -121.50]	
Test of $\theta_i = \theta_j$: Q(7) = 178.30, p = 0.00								
Test of θ = 0: z = -4.30, p = 0.00								
**Sublingual**								
El Maraghy 2023	20	336	160.9	20	445	213.9	-109.00 [-226.31, 8.31]	5.67
Heterogeneity: τ^2 = 0.00, I^2 = .%, H^2 = .							-109.00 [-226.31, 8.31]	
Test of $\theta_i = \theta_j$: Q(0) = -0.00, p = .								
Test of θ = 0: z = -1.82, p = 0.07								
**Vaginal**								
Abdulhakeem 2023	40	462.08	15.43	40	661.82	12.85	-199.74 [-205.96, -193.52]	9.51
Alhalaby 2021	25	308	32.66	40	404.4	87	-96.40 [-132.06, -60.74]	8.96
Celik 2003	13	472	77	12	621	121	-149.00 [-227.84, -70.16]	7.28
Moses 2018	23	406.03	381.82	24	416.51	418.16	-10.48 [-239.70, 218.74]	2.64
Niroomaand 2015	40	458	287	40	696	411	-238.00 [-393.35, -82.65]	4.34
Nnagbo 2023	63	522.6	127.91	63	583.5	156.2	-60.90 [-110.75, -11.05]	8.48
Heterogeneity: τ^2 = 5597.78, I^2 = 92.12%, H^2 = 12.69							-131.57 [-201.67, -61.47]	
Test of $\theta_i = \theta_j$: Q(5) = 63.44, p = 0.00								
Test of θ = 0: z = -3.68, p = 0.00								
**Overall**							-180.20 [-224.04, -136.35]	
Heterogeneity: τ^2 = 5252.75, I^2 = 95.93%, H^2 = 24.58								
Test of $\theta_i = \theta_j$: Q(14) = 344.07, p = 0.00								
Test of θ = 0: z = -8.05, p = 0.00								
Test of group differences: $Q_b(2)$ = 2.73, p = 0.26								

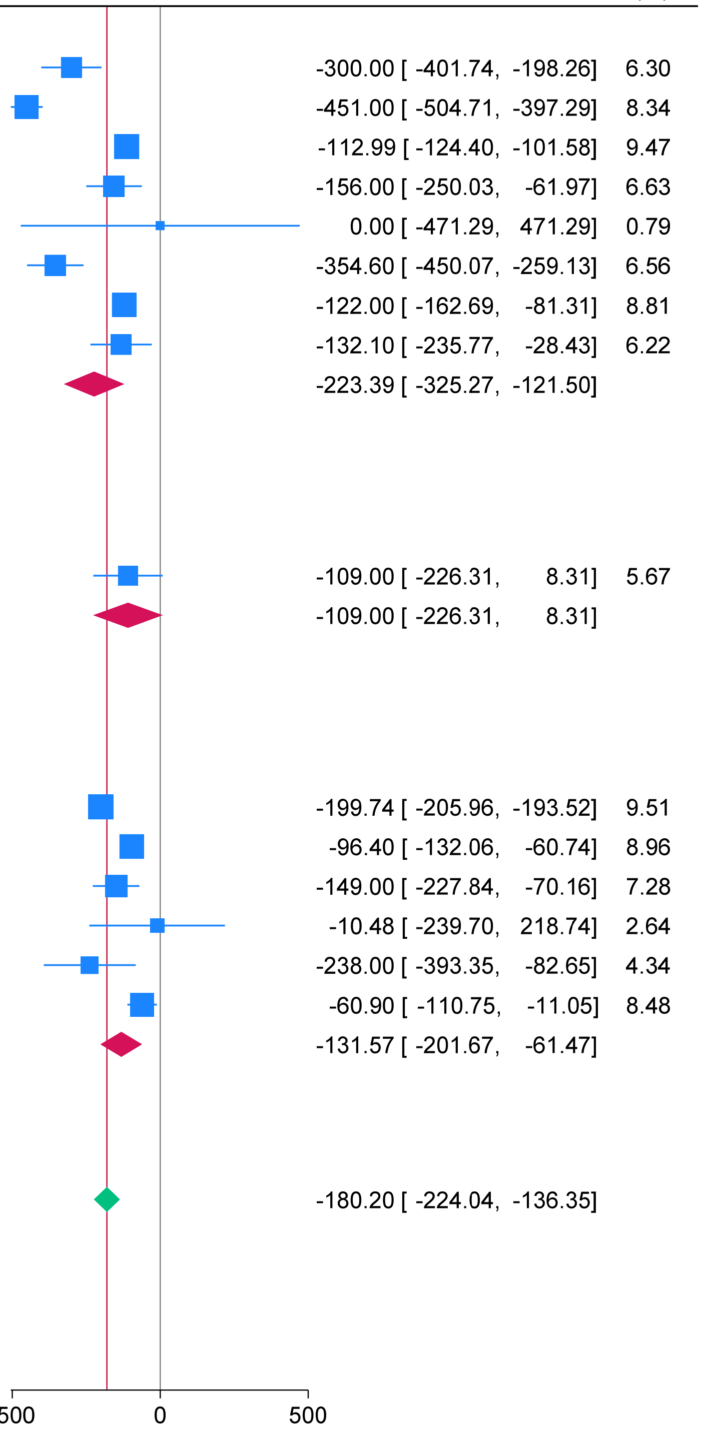

[B]

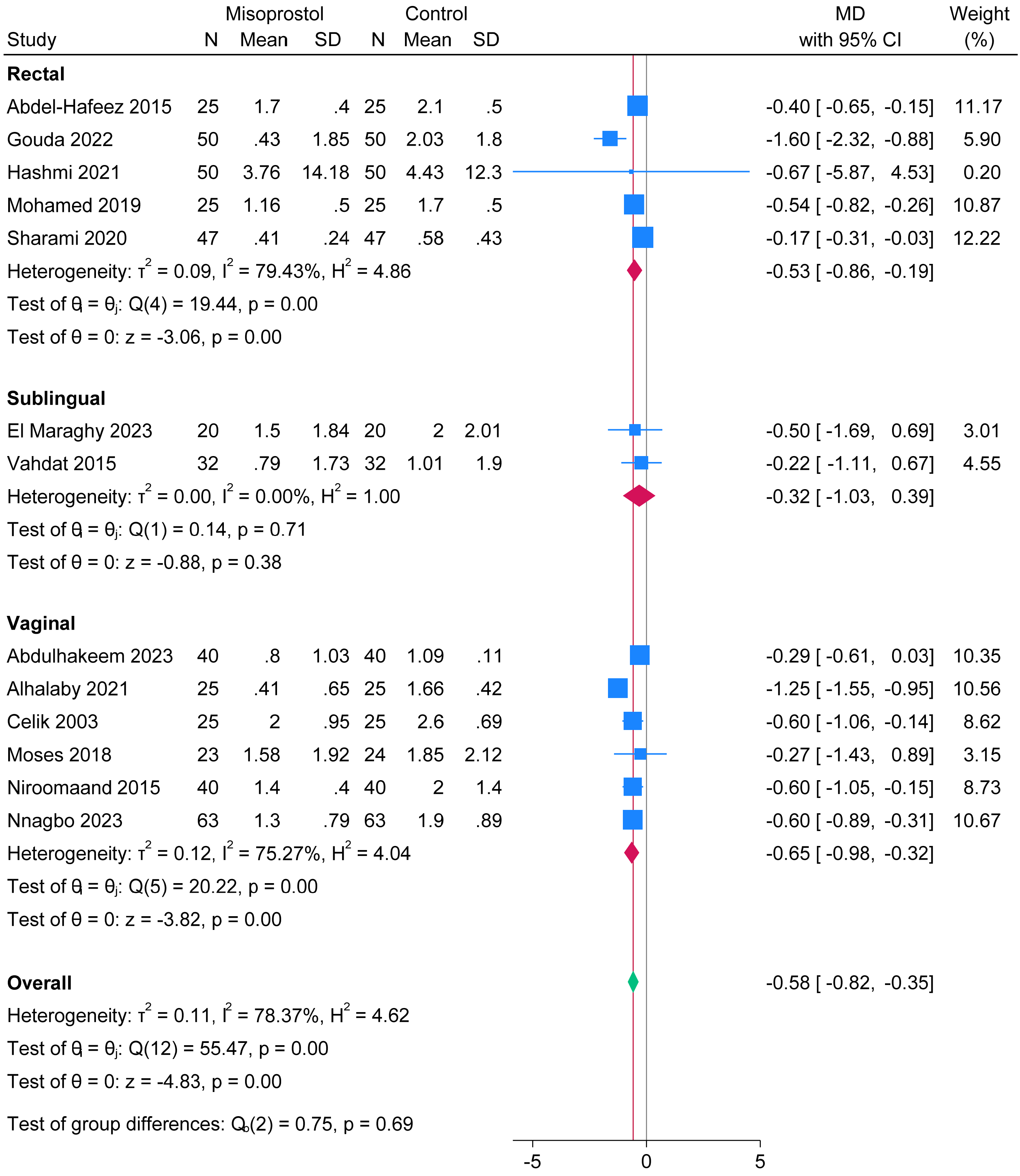
Misoprostol
Control
Study N Mean SD N Mean SD
MD with 95% CI
Weight (%)
Rectal
Abdel-Hafeez 2015 25 1.7 .4 25 2.1 .5 -0.40 [-0.65, -0.15] 11.17
Gouda 2022 50 .43 1.85 50 2.03 1.8 -1.60 [-2.32, -0.88] 5.90
Hashmi 2021 50 3.76 14.18 50 4.43 12.3 -0.67 [-5.87, 4.53] 0.20
Mohamed 2019 25 1.16 .5 25 1.7 .5 -0.54 [-0.82, -0.26] 10.87
Sharami 2020 47 .41 .24 47 .58 .43 -0.17 [-0.31, -0.03] 12.22
Heterogeneity: τ^2 = 0.09, I^2 = 79.43%, H^2 = 4.86 -0.53 [-0.86, -0.19]
Test of $\theta_i = \theta_j$: Q(4) = 19.44, p = 0.00
Test of θ = 0: z = -3.06, p = 0.00
Sublingual
El Maraghy 2023 20 1.5 1.84 20 2 2.01 -0.50 [-1.69, 0.69] 3.01
Vahdat 2015 32 .79 1.73 32 1.01 1.9 -0.22 [-1.11, 0.67] 4.55
Heterogeneity: τ^2 = 0.00, I^2 = 0.00%, H^2 = 1.00 -0.32 [-1.03, 0.39]
Test of $\theta_i = \theta_j$: Q(1) = 0.14, p = 0.71
Test of θ = 0: z = -0.88, p = 0.38
Vaginal
Abdulhakeem 2023 40 .8 1.03 40 1.09 .11 -0.29 [-0.61, 0.03] 10.35
Alhalaby 2021 25 .41 .65 25 1.66 .42 -1.25 [-1.55, -0.95] 10.56
Celik 2003 25 2 .95 25 2.6 .69 -0.60 [-1.06, -0.14] 8.62
Moses 2018 23 1.58 1.92 24 1.85 2.12 -0.27 [-1.43, 0.89] 3.15
Niroomaand 2015 40 1.4 .4 40 2 1.4 -0.60 [-1.05, -0.15] 8.73
Nnagbo 2023 63 1.3 .79 63 1.9 .89 -0.60 [-0.89, -0.31] 10.67
Heterogeneity: τ^2 = 0.12, I^2 = 75.27%, H^2 = 4.04 -0.65 [-0.98, -0.32]
Test of $\theta_i = \theta_j$: Q(5) = 20.22, p = 0.00
Test of θ = 0: z = -3.82, p = 0.00
Overall -0.58 [-0.82, -0.35]
Heterogeneity: τ^2 = 0.11, I^2 = 78.37%, H^2 = 4.62
Test of $\theta_i = \theta_j$: Q(12) = 55.47, p = 0.00
Test of θ = 0: z = -4.83, p = 0.00
Test of group differences: $Q_b(2)$ = 0.75, p = 0.69
-5
0
5

[C]

Study	Misoprostol Yes	Misoprostol No	Control Yes	Control No	RR with 95% CI	Weight (%)
**Rectal**						
Abdel-Hafeez 2015	6	19	9	16	0.67 [0.28, 1.59]	18.84
Gouda 2022	0	50	36	14	0.01 [0.00, 0.22]	1.99
Hashmi 2021	2	48	3	47	0.67 [0.12, 3.82]	4.93
Khan 2020	1	24	5	20	0.20 [0.03, 1.59]	3.51
Mohamed 2019	1	24	4	21	0.25 [0.03, 2.08]	3.36
Rashed 2014	0	20	2	16	0.18 [0.01, 3.54]	1.72
Sharami 2020	4	43	5	42	0.80 [0.23, 2.80]	9.45
Heterogeneity: $\tau^2 = 0.37$, $I^2 = 34.37\%$, $H^2 = 1.52$					0.38 [0.17, 0.85]	
Test of $\theta_i = \theta_j$: Q(6) = 9.14, p = 0.17						
Test of $\theta = 0$: z = -2.36, p = 0.02						
**Sublingual**						
El Maraghy 2023	0	20	1	19	0.33 [0.01, 7.72]	1.54
Vahdat 2015	5	27	8	24	0.62 [0.23, 1.71]	14.42
Heterogeneity: $\tau^2 = 0.00$, $I^2 = 0.00\%$, $H^2 = 1.00$					0.59 [0.23, 1.53]	
Test of $\theta_i = \theta_j$: Q(1) = 0.14, p = 0.71						
Test of $\theta = 0$: z = -1.08, p = 0.28						
**Vaginal**						
Abdulhakeem 2023	5	35	12	28	0.42 [0.16, 1.07]	16.13
Celik 2003	2	11	4	8	0.46 [0.10, 2.08]	6.60
Moses 2018	4	19	13	11	0.32 [0.12, 0.84]	15.59
Niroomaand 2015	0	40	9	31	0.05 [0.00, 0.87]	1.92
Heterogeneity: $\tau^2 = 0.00$, $I^2 = 0.00\%$, $H^2 = 1.00$					0.35 [0.19, 0.64]	
Test of $\theta_i = \theta_j$: Q(3) = 2.04, p = 0.56						
Test of $\theta = 0$: z = -3.44, p = 0.00						
**Overall**					0.43 [0.29, 0.63]	
Heterogeneity: $\tau^2 = 0.01$, $I^2 = 2.47\%$, $H^2 = 1.03$						
Test of $\theta_i = \theta_j$: Q(12) = 12.30, p = 0.42						
Test of $\theta = 0$: z = -4.24, p = 0.00						
Test of group differences: $Q_b(2) = 0.85$, p = 0.65						

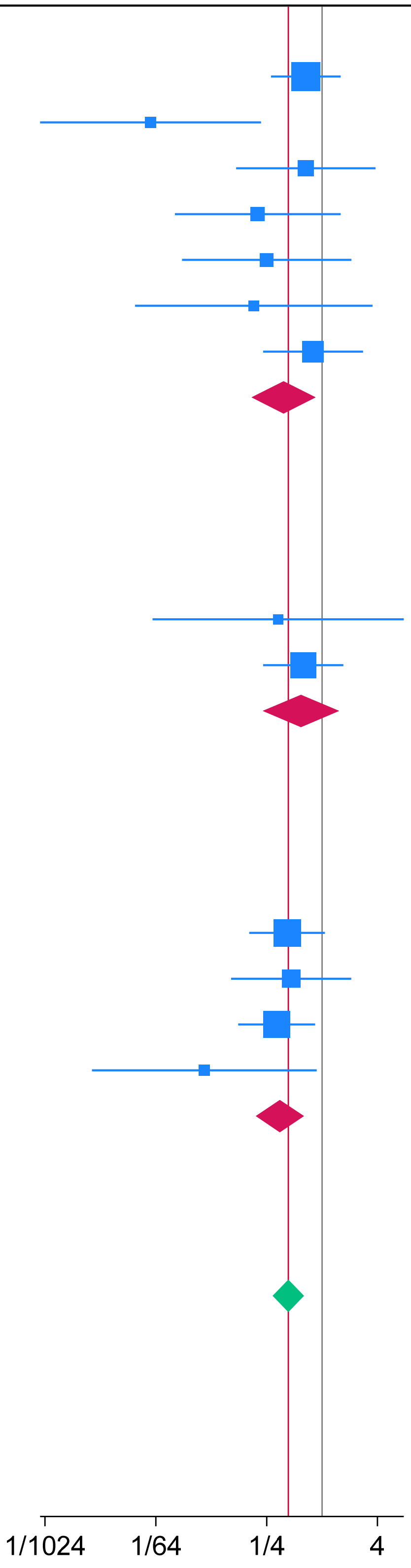

[D]

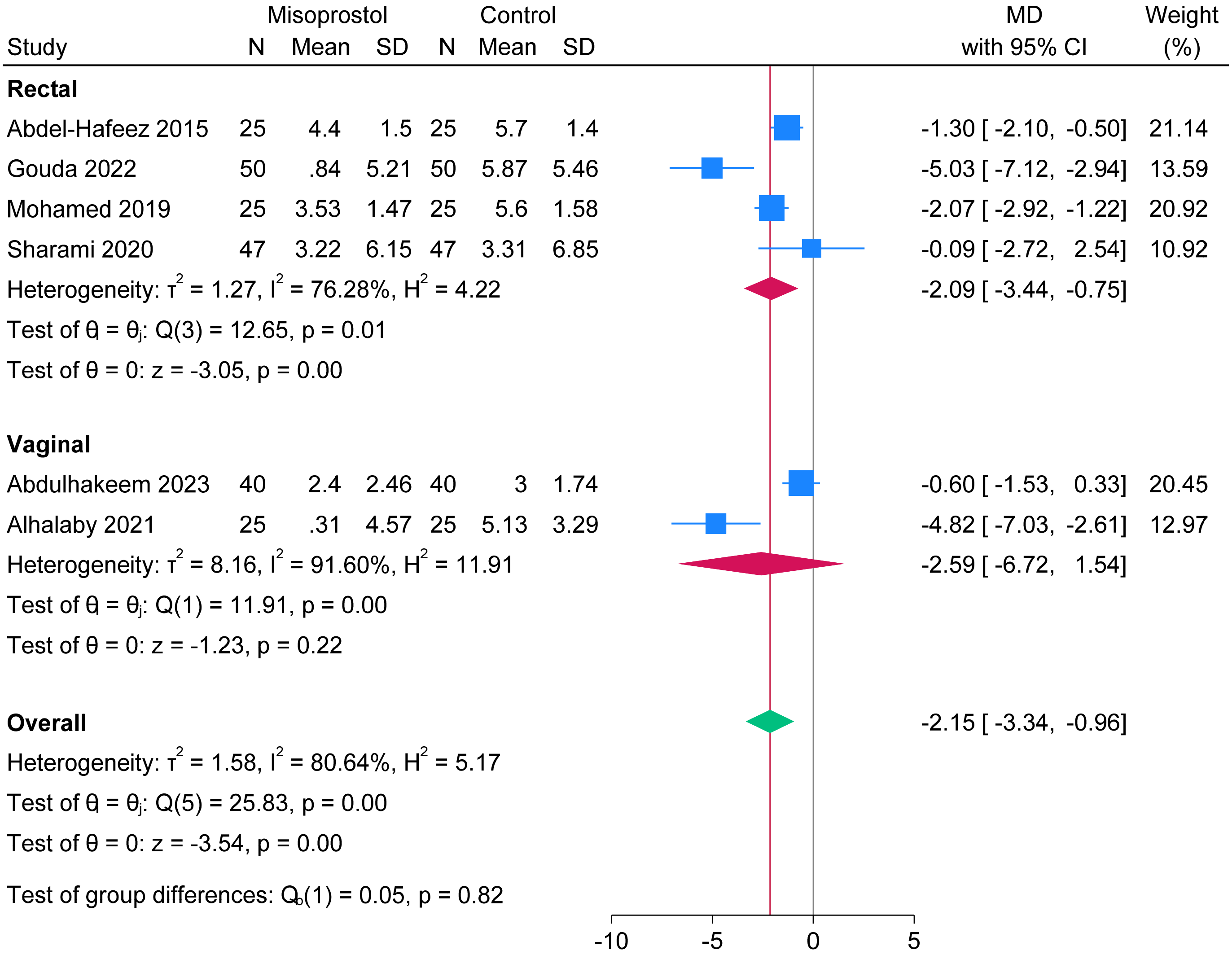
Misoprostol
Control
Study
N
Mean
SD
N
Mean
SD
MD
with 95% CI
Weight
(%)
Rectal
Abdel-Hafeez 2015 25 4.4 1.5 25 5.7 1.4 -1.30 [-2.10, -0.50] 21.14
Gouda 2022 50 .84 5.21 50 5.87 5.46 -5.03 [-7.12, -2.94] 13.59
Mohamed 2019 25 3.53 1.47 25 5.6 1.58 -2.07 [-2.92, -1.22] 20.92
Sharami 2020 47 3.22 6.15 47 3.31 6.85 -0.09 [-2.72, 2.54] 10.92
Heterogeneity: τ² = 1.27, I² = 76.28%, H² = 4.22 -2.09 [-3.44, -0.75]
Test of θi = θj: Q(3) = 12.65, p = 0.01
Test of θ = 0: z = -3.05, p = 0.00
Vaginal
Abdulhakeem 2023 40 2.4 2.46 40 3 1.74 -0.60 [-1.53, 0.33] 20.45
Alhalaby 2021 25 .31 4.57 25 5.13 3.29 -4.82 [-7.03, -2.61] 12.97
Heterogeneity: τ² = 8.16, I² = 91.60%, H² = 11.91 -2.59 [-6.72, 1.54]
Test of θi = θj: Q(1) = 11.91, p = 0.00
Test of θ = 0: z = -1.23, p = 0.22
Overall -2.15 [-3.34, -0.96]
Heterogeneity: τ² = 1.58, I² = 80.64%, H² = 5.17
Test of θi = θj: Q(5) = 25.83, p = 0.00
Test of θ = 0: z = -3.54, p = 0.00
Test of group differences: Qb(1) = 0.05, p = 0.82
-10
-5
0
5

[E]

Study	Misoprostol N	Misoprostol Mean	Misoprostol SD	Control N	Control Mean	Control SD	MD with 95% CI	Weight (%)
**Rectal**								
Abdel-Hafeez 2015	25	76.8	15.8	25	94.8	22.8	-18.00 [-28.87, -7.13]	9.02
Gouda 2022	50	68.5	10.98	50	85.7	24.45	-17.20 [-24.63, -9.77]	10.23
Maneerat 2019	14	132.9	38.77	15	139	50.01	-6.10 [-38.84, 26.64]	3.24
Mohamed 2019	25	70.4	11.3	25	84.6	21.2	-14.20 [-23.62, -4.78]	9.55
Sharami 2020	47	17.63	85.13	47	68.51	17.41	-50.88 [-75.72, -26.04]	4.66
Heterogeneity: $\tau^2 = 37.08$, $I^2 = 49.27\%$, $H^2 = 1.97$							-18.85 [-26.91, -10.78]	
Test of $\theta_i = \theta_j$: Q(4) = 7.89, p = 0.10								
Test of θ = 0: z = -4.58, p = 0.00								
**Sublingual**								
El Maraghy 2023	20	29.1	6.5	20	33.2	7.2	-4.10 [-8.35, 0.15]	11.10
Vahdat 2015	32	78.28	17.94	32	82.34	21.84	-4.06 [-13.85, 5.73]	9.41
Heterogeneity: $\tau^2 = 0.00$, $I^2 = 0.00\%$, $H^2 = 1.00$							-4.09 [-7.99, -0.19]	
Test of $\theta_i = \theta_j$: Q(1) = 0.00, p = 0.99								
Test of θ = 0: z = -2.06, p = 0.04								
**Vaginal**								
Abdulhakeem 2023	40	67.77	3.78	40	66.75	6.47	1.02 [-1.30, 3.34]	11.44
Alhalaby 2021	25	56.8	3.12	25	78.6	10.6	-21.80 [-26.13, -17.47]	11.09
Celik 2003	13	48.5	7.4	12	58	8.8	-9.50 [-15.86, -3.14]	10.56
Moses 2018	23	108.17	71.88	24	110.83	49.42	-2.66 [-37.80, 32.48]	2.92
Niroomaand 2015	40	106	29	40	127	47	-21.00 [-38.11, -3.89]	6.79
Heterogeneity: $\tau^2 = 174.86$, $I^2 = 95.50\%$, $H^2 = 22.22$							-11.30 [-24.33, 1.73]	
Test of $\theta_i = \theta_j$: Q(4) = 88.86, p = 0.00								
Test of θ = 0: z = -1.70, p = 0.09								
**Overall**							-12.95 [-19.89, -6.01]	
Heterogeneity: $\tau^2 = 108.14$, $I^2 = 90.84\%$, $H^2 = 10.92$								
Test of $\theta_i = \theta_j$: Q(11) = 120.15, p = 0.00								
Test of θ = 0: z = -3.66, p = 0.00								
Test of group differences: $Q_b(2) = 10.82$, p = 0.00								

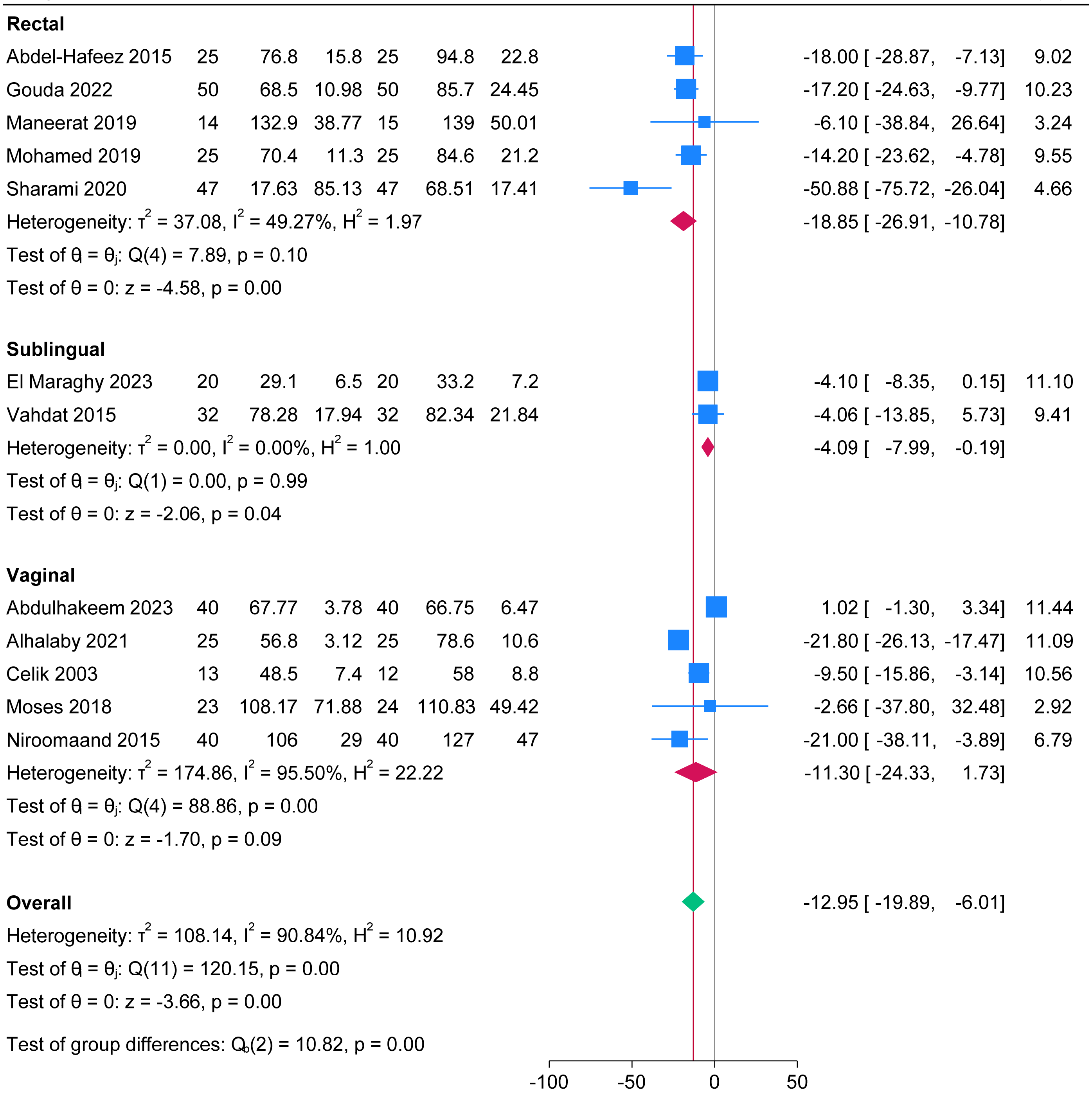

Supplementary Figure S2. Sensitivity analysis of the primary endpoints: **[A]** intraoperative blood loss (ml), **[B]** mean hemoglobin drop (mg/dl), and **[C]** rate of perioperative blood transfusion (%).

[A]

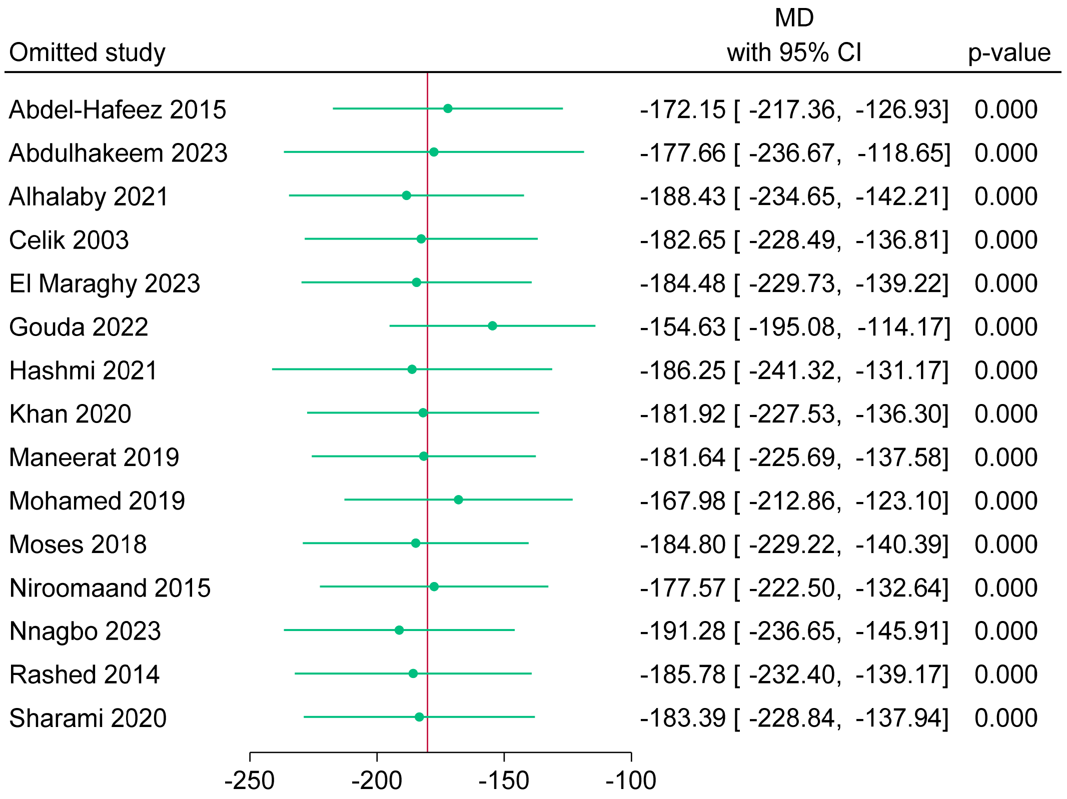

[B]

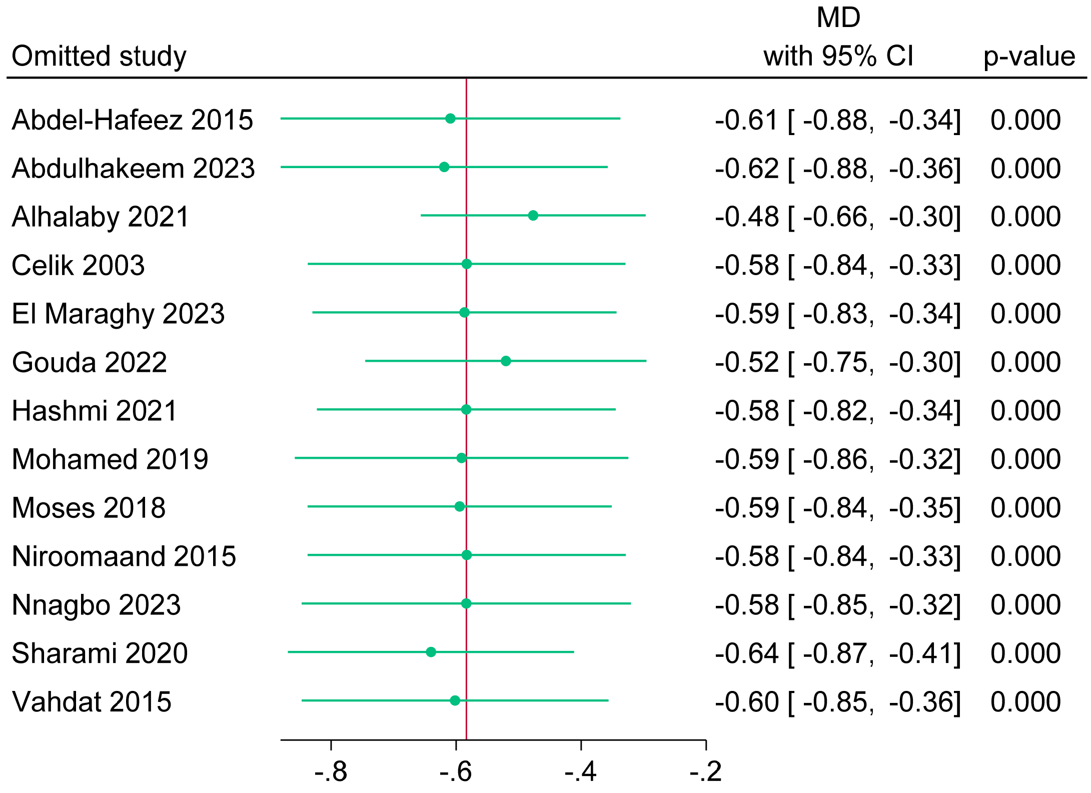

[C]

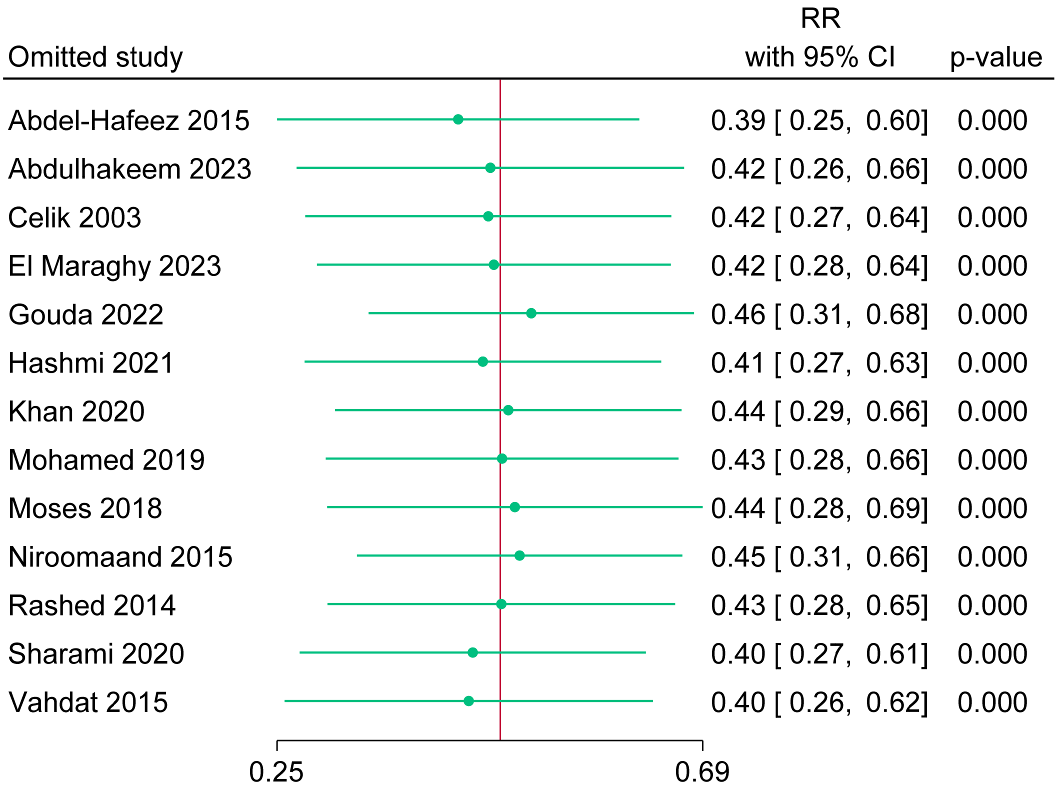

Omitted study
RR
with 95% CI
p-value
Abdel-Hafeez 2015
0.39 [0.25, 0.60]
0.000
Abdulhakeem 2023
0.42 [0.26, 0.66]
0.000
Celik 2003
0.42 [0.27, 0.64]
0.000
El Maraghy 2023
0.42 [0.28, 0.64]
0.000
Gouda 2022
0.46 [0.31, 0.68]
0.000
Hashmi 2021
0.41 [0.27, 0.63]
0.000
Khan 2020
0.44 [0.29, 0.66]
0.000
Mohamed 2019
0.43 [0.28, 0.66]
0.000
Moses 2018
0.44 [0.28, 0.69]
0.000
Niroomaand 2015
0.45 [0.31, 0.66]
0.000
Rashed 2014
0.43 [0.28, 0.65]
0.000
Sharami 2020
0.40 [0.27, 0.61]
0.000
Vahdat 2015
0.40 [0.26, 0.62]
0.000
0.25
0.69

Supplementary Figure S3. Sensitivity analysis of the secondary endpoints: **[A]** hematocrit drop (%), **[B]** operative time (min), **[C]** hospital stay (day), and **[D]** postoperative fever (%).

[A]

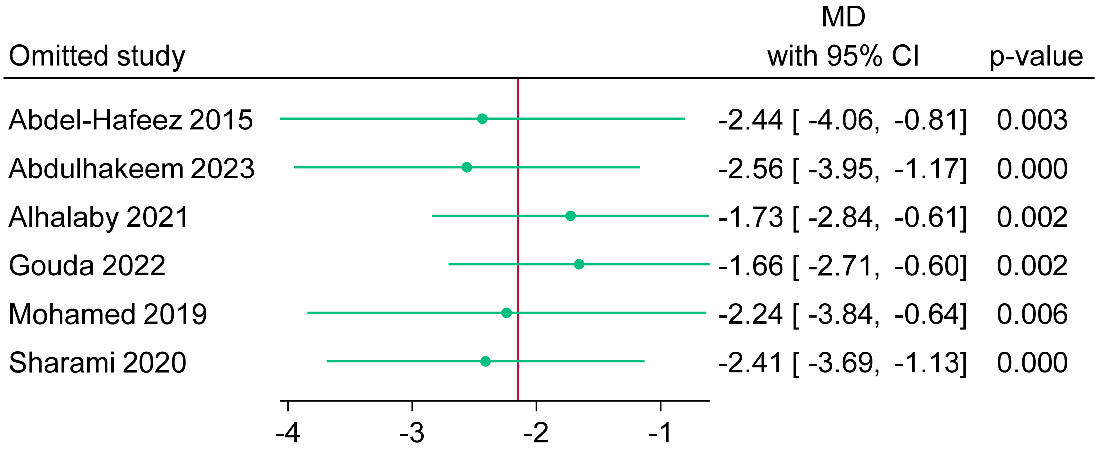

[B]

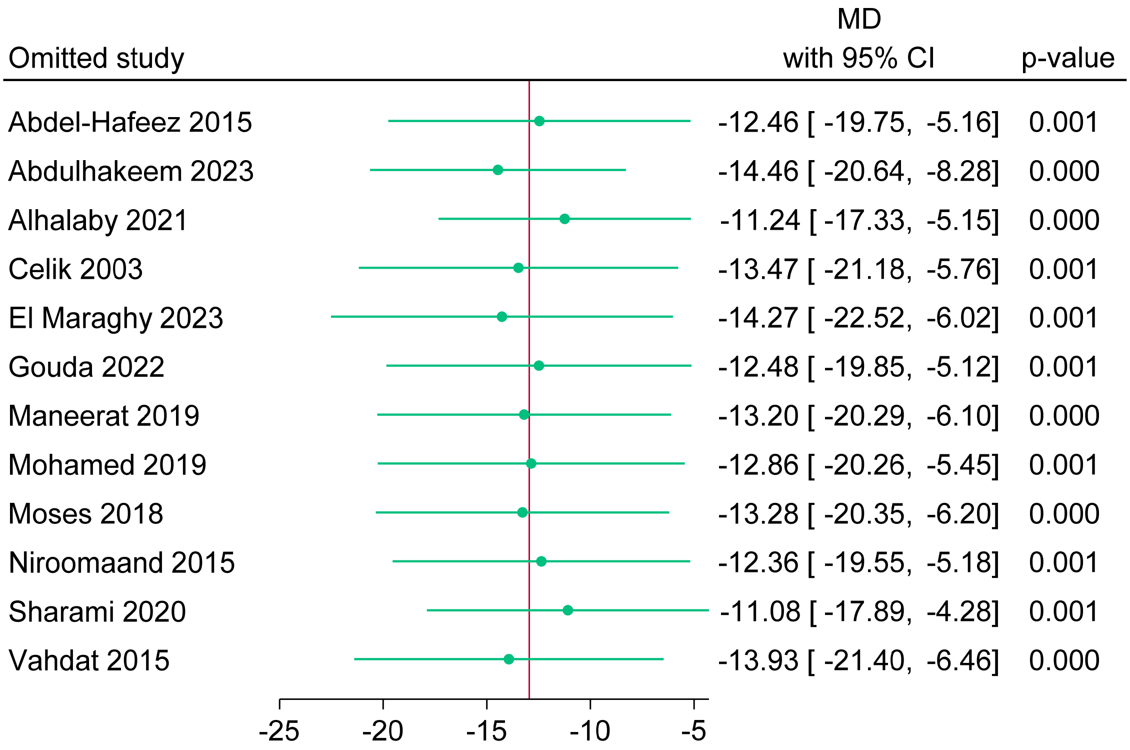

[C]

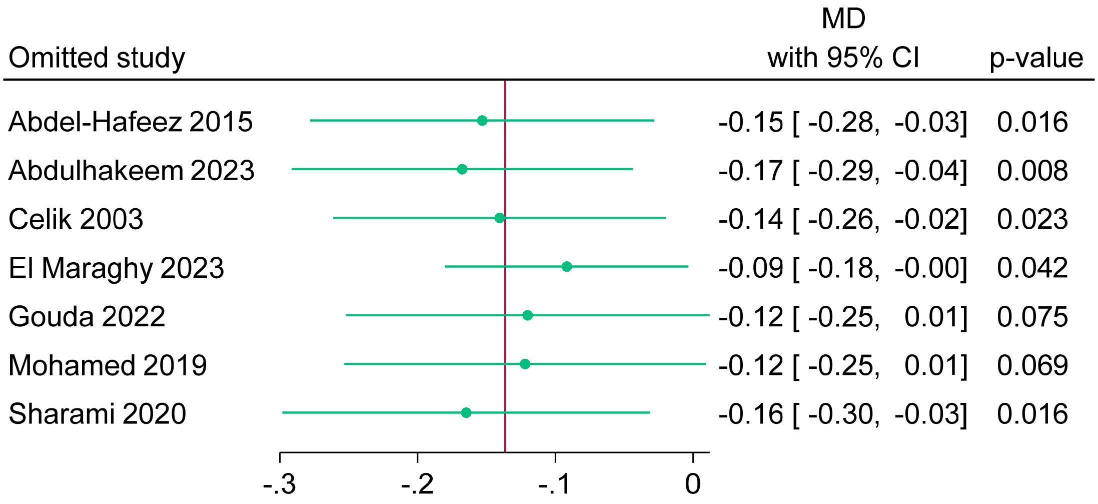
MD
Omitted study
with 95% CI
p-value
Abdel-Hafeez 2015
-0.15 [-0.28, -0.03]
0.016
Abdulhakeem 2023
-0.17 [-0.29, -0.04]
0.008
Celik 2003
-0.14 [-0.26, -0.02]
0.023
El Maraghy 2023
-0.09 [-0.18, -0.00]
0.042
Gouda 2022
-0.12 [-0.25, 0.01]
0.075
Mohamed 2019
-0.12 [-0.25, 0.01]
0.069
Sharami 2020
-0.16 [-0.30, -0.03]
0.016
-.3
-.2
-.1
0

[D]

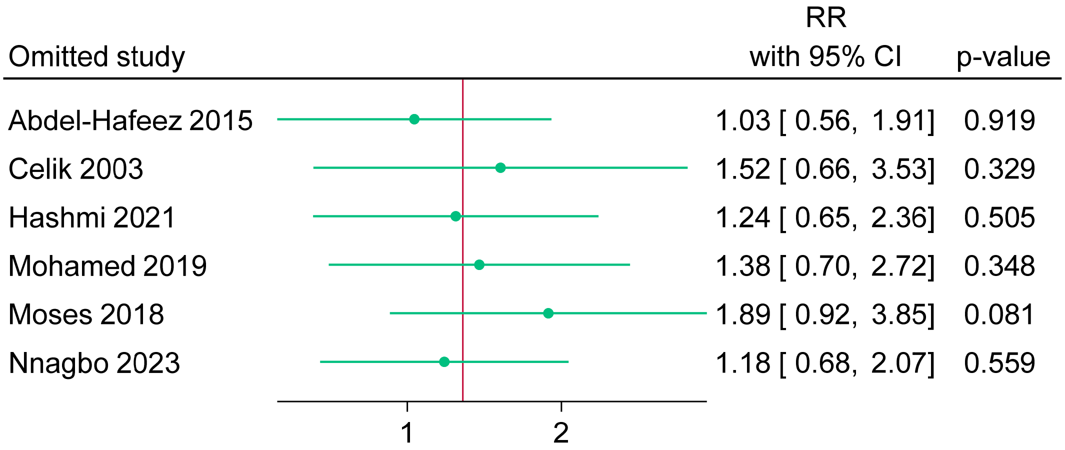
RR
Omitted study
with 95% CI
p-value
Abdel-Hafeez 2015
1.03 [0.56, 1.91]
0.919
Celik 2003
1.52 [0.66, 3.53]
0.329
Hashmi 2021
1.24 [0.65, 2.36]
0.505
Mohamed 2019
1.38 [0.70, 2.72]
0.348
Moses 2018
1.89 [0.92, 3.85]
0.081
Nnagbo 2023
1.18 [0.68, 2.07]
0.559
1
2

Supplementary Figure S4. Qualitative publication bias analysis according to funnel plots: **[A]** intraoperative blood loss (ml), **[B]** mean hemoglobin drop (mg/dl), **[C]** rate of perioperative blood transfusion (%), and **[D]** operative time (min).

[A]

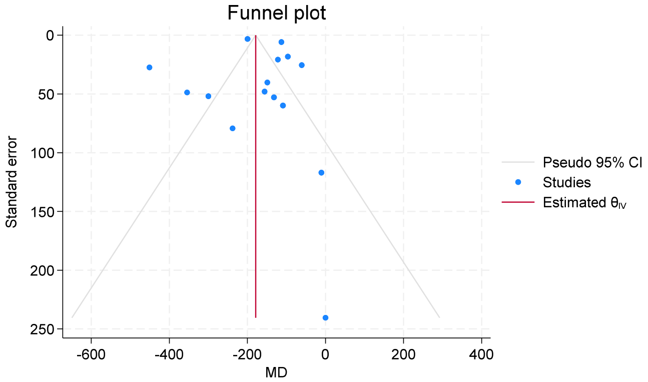

[B]

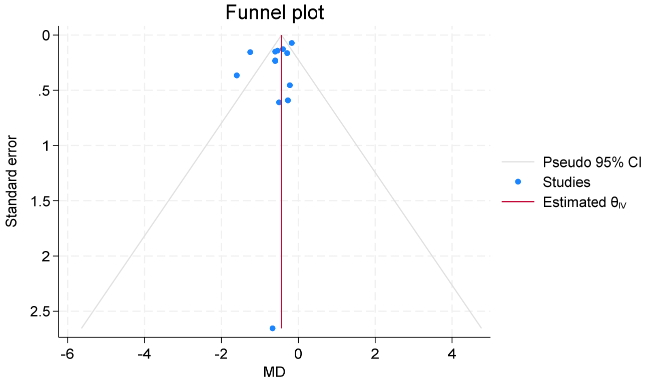

[C]

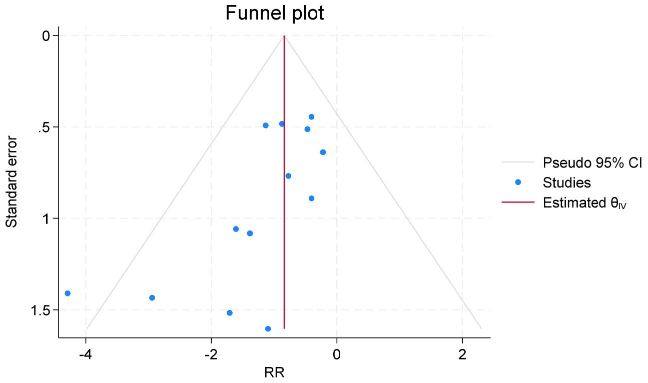

[D]

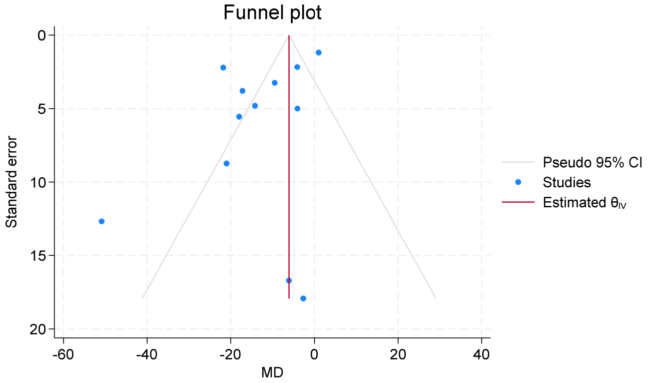

Supplement: Supplementary file 1 [file jcm-13-06356-s001.zip › Supplementary Figures_10-15-24.pdf]
