# Supplementary material for: Clinical Efficacy and Safety of Misoprostol During Abdominal Myomectomy: An Updated Systematic Review and Meta-Analysis of 16 Randomized Controlled Trials
Source: J Clin Med. 2024 Oct 24;13(21):6356. doi: 10.3390/jcm13216356 (PMC11546417; doi:10.3390/jcm13216356)
Supplement: Supplementary file 1 [file jcm-13-06356-s001.zip › Supplementary Tables_10-15-24.pdf]

Supplementary Table S1. The search strategy used in all databases.

**PubMed**<br>All Fields: (PGE1 OR "prostaglandin E1" OR misotac OR cytotec OR misoprostol) AND (myomectomy) AND (open OR abdominal OR laparotomy)
**Scopus**<br>TITLE-ABS-KEY ((pge1 OR "prostaglandin e1" OR misotac OR cytotec OR misoprostol) AND (myomectomy) AND (open OR abdominal OR laparotomy))
**Web of Science**<br>All Fields: ('pge1'/exp OR pge1 OR 'prostaglandin e1'/exp OR 'prostaglandin e1' OR 'misotac'/exp OR misotac OR 'cytotec'/exp OR cytotec OR 'misoprostol'/exp OR misoprostol) AND ('myomectomy'/exp OR myomectomy) AND (open OR abdominal OR 'laparotomy'/exp OR laparotomy)
**Embase**<br>Broad search: ('carbetocin'/exp OR carbetocin OR 'duratocin'/exp OR duratocin OR papal) AND ('myomectomy'/exp OR myomectomy)
**Cochrane Central Register of Controlled Trials (CENTRAL)**<br>Title Abstract Keyword: (PGE1 OR "prostaglandin E1" OR misotac OR cytotec OR misoprostol) AND (myomectomy) AND (open OR abdominal OR laparotomy)
**Google Scholar**<br>All Fields: (PGE1 OR "prostaglandin E1" OR misotac OR cytotec OR misoprostol) AND (myomectomy) AND (open OR abdominal OR laparotomy)

Supplementary Table S2. Quantitative publication bias analysis according to Egger's regression test.

**Endpoint**	**p value**
Intraoperative blood loss	0.7308
Hemoglobin drop	0.7018
Perioperative blood transfusion	0.2044
Operative time	0.2336

Supplementary Table S3. Summary of the certainty of evidence according to the GRADE approach.

Endpoint	Certainty Assessment							Overall certainty
	# of studies	Study design	Risk of bias	Inconsistency[a]	Indirectness	Imprecision[b]	Publication bias	
**Intraoperative blood loss**	15	Randomized trials	Not serious	Serious	Not serious	Not serious	Undetected	⊕⊕⊕◯ Moderate
**Hemoglobin drop**	13	Randomized trials	Not serious	Serious	Not serious	Not serious	Undetected	⊕⊕⊕◯ Moderate
**Blood transfusion**	13	Randomized trials	Not serious	Not serious	Not serious	Not serious	Undetected	⊕⊕⊕⊕ High
**Hematocrit drop**	6	Randomized trials	Not serious	Serious	Not serious	serious	.	⊕⊕◯◯ Low
**Operative time**	12	Randomized trials	Not serious	Serious	Not serious	Not serious	Undetected	⊕⊕⊕◯ Moderate
**Hospital stay**	7	Randomized trials	Not serious	Not serious	Not serious	serious	.	⊕⊕⊕◯ Moderate
**Postoperative fever**	6	Randomized trials	Not serious	Not serious	Not serious	serious	.	⊕⊕⊕◯ Moderate

[a] Inconsistency was judged as serious when the between-study heterogeneity was significant (I^2 statistic>50%).
[b] Imprecision was judged as serious because of the small number of studies and sample sizes.
